# Modifiable Risk Factors in Atrial Fibrillation: Clinical Implications and Pathophysiological Insights

**DOI:** 10.1111/anec.70171

**Published:** 2026-03-02

**Authors:** Chamath Jagoda

**Affiliations:** ^1^ Macquarie Medical School, Faculty of Medicine, Health and Human Sciences Macquarie University Sydney Australia

## Abstract

**Background:**

Atrial fibrillation (AF) is the most common sustained arrhythmia worldwide, and its growing prevalence is increasingly driven by modifiable lifestyle and clinical factors. Understanding how obesity, hypertension, diabetes, obstructive sleep apnoea (OSA), alcohol intake, and cardiorespiratory fitness each contribute to AF onset, progression, and recurrence is essential for developing comprehensive prevention and treatment strategies.

**Methods:**

We performed a narrative review of the available literature assessing six key modifiable risk factors for AF. Evidence was synthesized regarding each factor's pathophysiological effects on atrial structure and electrophysiology, as well as the impact of targeted interventions on arrhythmia burden and procedural outcomes.

**Results:**

Every 1 kg/m^2^ increase in body mass index (BMI) raises AF risk by 50%, with sustained weight loss reversing atrial remodeling and improving arrhythmia‐free survival. Hypertension increases atrial pressure, electrical heterogeneity, and fibrosis; resistant cases may benefit from adjunctive renal denervation. Diabetes promotes oxidative stress, profibrotic signaling, and autonomic neuropathy, reducing cardioversion success and antiarrhythmic efficacy. OSA induces intermittent hypoxia and sympathetic surges, destabilizing atrial substrates; CPAP therapy reduces remodeling and halves recurrence after cardioversion or ablation. Alcohol exerts dose‐dependent ion‐channel alterations, structural changes, and autonomic disruption. Cardiorespiratory fitness shows a U‐shaped relationship: moderate exercise is protective, whereas endurance extremes and inactivity foster atrial stretch, fibrosis, and ectopic triggers. Across all factors, shared mechanisms of fibrosis, inflammation, and autonomic imbalance emerge as key therapeutic targets.

**Conclusions:**

Integrating risk‐factor modification into AF rhythm‐control protocols addresses fundamental disease mechanisms and enhances procedural efficacy, offering clinicians a framework for prevention and therapy.

## Graphical abstract

Modifiable cardiometabolic and lifestyle factors converge on shared atrial mechanisms, including fibrosis, autonomic imbalance, and electrical remodeling, to promote AF initiation and recurrence. Targeting these upstream drivers through structured risk‐factor management enhances rhythm‐control durability and improves long‐term clinical outcomes.

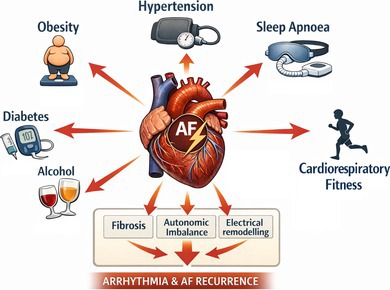

## 1 Introduction

Atrial fibrillation (AF) is the most common sustained cardiac arrhythmia with a threefold increase in prevalence observed over the past 50 years (Schnabel et al. 2015). Rates of hospitalization for AF have continued to outpace those for coronary disease and heart failure in the western world (Gallagher et al. 2019). The rising tide of AF appears to be largely driven by lifestyle risk factors that include obesity, hypertension, diabetes, obstructive sleep apnoea, cardiorespiratory fitness, and alcohol (Lau et al. 2017).

Moe et al. proposed that AF comprises electrical wavelets that continuously divide and coalesce across the atrial myocardium, enabling the arrhythmia to persist (Lee et al. 2020). Subsequent work has suggested that AF is initiated and maintained by an interaction between atrial triggers commonly emanating from the pulmonary veins and a complex atrial substrate characterized by regions of low voltage, fibrosis, and slowed conduction (Huang et al. 2022). Similar substrates have been identified across a spectrum of conditions that pre‐dispose to AF. Additionally, the degree of atrial remodeling continues to progress with persisting exposure to pro‐arrhythmic clinical states (Roka and Burright 2023). It is in this context that management of concomitant AF risk factors has emerged as a vital aspect of successful rhythm control strategies. Success rates of traditional rhythm control strategies have plateaued but appear to be vastly improved by management of AF risk factors (Gupta et al. 2013).

Despite well‐established links between rhythm control, risk‐factor management, and improved AF outcomes, the precise mechanisms driving these benefits remain ill‐defined, and the comparative contribution of individual risk factors is poorly understood. This review synthesizes the evidence on risk‐factor modification in AF and elucidates the pathophysiological mechanisms through which each intervention may confer clinical benefit.

## 2 Aim

This narrative review aims to synthesize evidence on major modifiable risk factors for AF, relating clinical outcomes (incident AF, progression, recurrence, and rhythm‐control outcomes) to the mechanistic pathways driving atrial structural and electrophysiological remodeling.

## 3 Obesity

### 3.1 Epidemiology

Observational studies demonstrate that obesity increases the risk of incident AF by 50%, with each 1‐unit rise in body mass index (BMI) conferring a 4% increase (Wang et al. 2004). Although this relationship is seen in both sexes, males are disproportionately affected: those with BMI > 40 face a 4.5‐fold higher AF risk compared with normal‐BMI individuals, whereas females show a two‐fold increase. The association is strongest among men under 65, and obesity also promotes progression from paroxysmal to persistent AF, implying a dose–response effect on atrial remodeling (Tsang et al. 2008). Conversely, AF risk in both men and women decreases as BMI falls (Ball et al. 2018). Increased height, weight, proportion of body fat, lean body mass and hip‐to‐waist circumference ratio each associate with AF predisposition. Although increased adiposity often coincides with higher lean mass, lean body mass remains an independent risk factor even after adjusting for height and body‐fat percentage. Obesity often occurs in the context of multiple concomitant risk factors and comorbidities such as hypertension, diabetes, obstructive sleep apnoea, myocardial infarction, valvular heart disease and heart failure, that associate closely with AF, making it challenging to delineate the direct causal relationship (Frost et al. 2014).

### 3.2 Long‐Term Weight Gain and AF Risk

Long‐term weight gain from an early point in life and fluctuations in BMI contribute to a cumulative increase in AF development. An increase in BMI of > 5 kg/m^2^ over 40 years is associated with a three‐fold higher risk of incident AF, highlighting the importance of the temporal pattern of weight gain when estimating individual risk (Feng et al. 2019a). Weight gain later in life confers greater AF risk than comparable gain earlier (Feng et al. 2019a), and even over a 5‐year period, those who gain weight have higher AF rates than those who maintain stable weight (Frederiksen et al. 2024). These observations suggest that early intervention to prevent obesity or overweight may be critical to reducing long‐term AF risk.

### 3.3 Current BMI and AF Burden, Recurrence and Progression

Among patients with established AF, a higher BMI correlates with greater symptom burden and severity, particularly in those undergoing attempted rhythm control (Chalazan et al. 2018). In a prospective, multi‐centre cohort of 2497 patients followed for 12 months, AF recurrence rates rose in parallel with BMI (Providência et al. 2019). Likewise, the Women's Health Study found that progression from paroxysmal to persistent AF was more likely at higher BMI, peaking during the first year after diagnosis with progression rates of 8.6%–15% (Sandhu et al. 2014).

### 3.4 Effect of Weight Loss on AF Burden and Recurrence

Multiple studies demonstrate that structured weight loss significantly reduces AF burden and symptom severity. In a randomized trial of 150 patients, Abed et al. found that a dedicated risk‐factor management clinic not only led to weight reduction but also improved AF symptom severity and burden, alongside better blood pressure, lipid profile, glycaemic control, and sleep‐apnoea severity (Abed et al. 2013). Among patients undergoing catheter ablation, risk‐factor modification independently predicted freedom from recurrent AF and reduced need for further rhythm‐control interventions (Pathak et al. 2014). The LEGACY cohort further showed that ≥ 10% weight loss was associated with a six‐fold increase in arrhythmia‐free survival, whereas weight fluctuations > 5% doubled the risk of recurrence, underscoring the importance of consistent weight loss (Pathak, Middeldorp, et al. 2015).

### 3.5 Reverse Remodeling and the Need for Sustained Management

Beyond symptomatic improvement, weight loss may reverse atrial pathology that sustains AF. The REVERSE‐AF study reported that participants achieving ≥ 10% weight loss reverted from persistent to paroxysmal AF, while those with lesser weight loss or weight gain progressed to more sustained forms (Middeldorp et al. 2018). These observations highlight the necessity of long‐term, sustainable weight management to preserve AF benefits and prevent recurrence driven by weight variability (Pathak, Middeldorp, et al. 2015).

### 3.6 Metabolically Healthy Obesity and AF

Metabolically healthy obesity, defined as obesity with normal triglycerides, glucose, blood pressure, waist circumference and high‐density lipoprotein, is variably linked to AF risk. In a population study of 47,870 participants, Feng et al. found that both metabolically healthy and unhealthy obesity were associated with a similar 1.6‐fold increase in incident AF compared with non‐obese individuals, implying that obesity per se drives arrhythmic risk supporting early weight‐reduction strategies (Feng et al. 2019b). In contrast, Lee et al., in a large retrospective cohort, reported that obesity alone increased AF risk by 20%, whereas metabolically unhealthy obesity, primarily driven by hypertension, increased risk by 40%, suggesting that metabolic dysfunction further augments obesity‐related AF risk (Lee et al. 2017).

### 3.7 Catheter Ablation for AF

Despite multiple approaches to AF ablation, single‐procedure success rates remain modest, particularly in persistent AF. Observational studies have linked higher body weight to poorer arrhythmia‐free outcomes (Sivasambu et al. 2018), and Danish registry data in 9188 patients demonstrate an incremental rise in post‐ablation recurrence with increasing BMI, prompting the hypothesis that pre‐ablation weight loss may optimize long‐term freedom from AF (Tønnesen et al. 2022).

Episodes of AF occurring within 2 weeks to 3 months after ablation, termed early recurrences, affect about 35% of patients and are associated with worse long‐term outcomes; however, roughly 30% of those with early recurrences remain asymptomatic in the longer term. In such patients, short‐term use of antiarrhythmic drugs may be preferable before considering repeat ablation (Oral et al. 2002).

Long‐term AF recurrences often reflect residual pulmonary vein conduction and are influenced by comorbid risk factors such as obesity, obstructive sleep apnoea, hypertension and diabetes which may perpetuate arrhythmogenic substrate (Jongnarangsin et al. 2008). One study reported a 7% annual recurrence rate, with 71% of patients' arrhythmia‐free for up to 5 years after pulmonary vein ablation, underlining the frequent need for repeat procedures (Tzou et al. 2010). In the DISCERN‐AF trial, the ratio of asymptomatic to symptomatic episodes rose from 1.1 to 3.7 post‐ablation, total arrhythmia burden fell by 86%, and 58% of patients achieved symptom‐free status at 18 months after an average of 1.4 procedures (Verma et al. 2013).

Higher BMI exerts a greater negative impact on persistent AF than on paroxysmal AF, although all phenotypes fare poorly once BMI ≥ 40 kg/m^2^ (Sivasambu et al. 2018). Post‐ablation data reveal a dose‐dependent decline in five‐year arrhythmia‐free survival with rising BMI, particularly ≥ 35 kg/m^2^, supporting that weight loss in this group improves outcomes (Winkle et al. 2017). Glover et al. found obese patients (BMI ≥ 30 kg/m^2^) had a 1.2‐fold higher recurrence risk at 12 months than overweight patients (BMI 25.5–29 kg/m^2^) (Bunch et al. 2016), while those ablated at lower BMIs showed significantly fewer recurrences over 3 years (Bunch et al. 2016). Adjunctive antiarrhythmic drug (AAD) use post‐ablation correlates with enhanced long‐term AF freedom compared to no AAD (De Maat et al. 2018). Moreover, in paroxysmal AF, catheter ablation achieved 66% arrhythmia freedom at 9 months versus 16% with AAD alone (Wilber et al. 2010).

### 3.8 Bariatric Surgery and AF

In a Swedish cohort of 4021 obese patients without prior AF, bariatric surgery (BS) was linked to a 29% lower risk of developing AF over a median follow‐up of 19 years compared with medical management (Jamaly et al. 2016). Younger age and higher baseline diastolic blood pressure amplified this benefit, suggesting that obesity‐related cardiac remodeling may be more reversible in younger patients and that those with hypertension derive greater antihypertensive effects from weight loss. Importantly, the reduction in AF risk was consistent across all BMI categories, indicating that the degree of obesity did not significantly alter the protective effect of surgery (Jamaly et al. 2016).

Among patients with established AF, those who underwent BS experienced a lower incidence of AF than medically managed controls (Lynch et al. 2019). After gastric bypass, mean weight loss of 25% yielded larger decreases in inflammatory markers, blood pressure, and AF burden than sleeve gastrectomy or gastric banding. Post‐surgery, 27% of gastric bypass patients shifted from persistent to paroxysmal AF (versus 25% for sleeve gastrectomy and 23% for gastric banding), and overall AF reversal occurred in 71%, 56%, and 50% of these groups, respectively (Donnellan, Wazni, Elshazly, et al. 2020). Patients with permanent or long‐standing persistent AF showed minimal benefit, with 89% exhibiting no change or reversal following surgery (Donnellan, Wazni, Elshazly, et al. 2020).

### 3.9 Combining Bariatric Surgery With Catheter Ablation

Morbidly obese patients who undergo BS before catheter ablation experience markedly lower AF recurrence than both non‐BS morbidly obese patients and non‐obese controls. Over 12 months post‐ablation, the BS cohort maintained a 10% recurrence rate versus 31% in morbidly obese patients without prior BS (Donnellan, Wazni, Kanj, Hussein, et al. 2019). Donnellan et al. further demonstrated a three‐fold reduction in AF recurrence for BS patients and noted that those without BS required more repeat ablations to manage their arrhythmia (Donnellan, Wazni, Kanj, Hussein, et al. 2019).

Weight change prior to ablation also significantly affects outcomes: patients who gained weight before the procedure had a 78% recurrence rate, while those who lost more than 10% of their body weight saw recurrence drop to 33% (Donnellan, Wazni, Kanj, Baranowski, et al. 2019). These findings underline that pre‐ablation weight loss, whether through BS or other means, can align AF recurrence rates in morbidly obese individuals with those of non‐obese patients.

### 3.10 Cardioversion and AF

Direct current cardioversion (DCC) achieves an immediate success rate of approximately 86% in patients with AF. However, both longer arrhythmia duration and higher body weight reduce the likelihood of initial success (Elhendy et al. 2002). Recurrence after DCC is common, with early return of AF most strongly predicted by the duration of the arrhythmia before cardioversion: episodes terminating after < 3 h carry a three‐fold higher risk of early recurrence (Schwartzman et al. 2002).

## 4 Hypertension

### 4.1 Epidemiology

Hypertension is highly prevalent in AF, 60%–80% AF patients carry the diagnosis (Nabauer et al. 2009), and accounts for over 20% of incident AF in the Atherosclerosis Risk in Communities cohort (Huxley, Lopez, et al. 2011). In the Framingham Heart Study of 4731 participants, hypertension conferred a 50% higher AF risk in men and 40% in women (Benjamin et al. 1994). Even pre‐hypertension (120–139/80–89 mmHg) elevated AF risk by 80% compared with normotension (O'Neal et al. 2015). Systolic blood pressure is a stronger predictor of incident AF than diastolic pressure in both sexes, with higher systolic ranges paralleling AF onset (Grundvold et al. 2012), although at certain systolic thresholds lower diastolic pressures may paradoxically increase new‐onset AF risk (Larstorp et al. 2012).

### 4.2 Pulse Pressure as a Predictor of AF

Pulse pressure (PP), an indirect marker of arterial stiffness, is a strong prognosticator for incident AF (Mitchell et al. 2007). In direct analyses, PP outperforms systolic blood pressure (SBP), diastolic blood pressure (DBP), and mean arterial pressure as a predictor of new‐onset AF, underscoring the role of arterial stiffness in AF pathogenesis (Larstorp et al. 2012; Mitchell et al. 2007).

### 4.3 Systolic and Diastolic Blood Pressure: J‐Curve Versus Linear Relationships

Observational data suggest a J‐shaped association between SBP and AF risk: individuals with SBP < 120 mmHg show raised AF incidence, while those with SBP ≥ 150 mmHg have around double the risk compared to SBP 120–129 mmHg, implying low SBP may reflect comorbid conditions that predispose to AF; beyond 120 mmHg, risk then increases linearly (Thomas et al. 2008). However, most cohort studies favor an approximately linear SBP–AF relationship. For example, the Women's Health Study found no J‐curve: women with SBP 130–139 mmHg or DBP ≈85 mmHg experienced a 28%–52% higher AF risk versus those with BP < 120/65 mmHg (Conen et al. 2009).

### 4.4 Antihypertensive Therapy in AF

Large trials comparing chlorthalidone, amlodipine, lisinopril, and other first‐line agents found no overall difference in new‐onset AF across most treatment arms; however, patients randomized to doxazosin experienced a higher incidence of AF than those on chlorthalidone (Haywood et al. 2009). Likewise, ACE‐inhibitor therapy alone did not significantly alter AF risk compared with other regimens, possibly reflecting the dominant influence of unmodifiable factors such as age and race (Haywood et al. 2009). A subsequent analysis directly comparing long‐term use of ACE inhibitors, ARBs, and β‐blockers against calcium‐channel blockers suggested that the former group may confer greater protection against AF development than calcium‐channel blockade alone (Schaer et al. 2010).

Antihypertensive strategies aimed at regression of ECG‐defined left ventricular hypertrophy also reduce AF risk: in one study, losartan therapy was linked to fewer incident AF events (Okin et al. 2006). Consistent with this, Heckbert et al. reported that hypertensive patients without heart failure treated with either an ACE inhibitor or an ARB had a lower AF incidence than those managed with diuretic monotherapy (Heckbert et al. 2009).

### 4.5 Impact of Hypertension Status on AF Ablation Outcomes

In a comparative cohort, uncontrolled hypertension was a strong predictor of post‐ablation AF recurrence. Patients with uncontrolled HTN had a 40.6% recurrence rate, versus 28.1% in those with controlled HTN and 25.7% in normotensive patients; no significant difference emerged between the latter two groups, underscoring the unique risk posed by poorly managed blood pressure (Santoro et al. 2015).

### 4.6 Renal Denervation as an Adjunct to AF Ablation

Renal denervation, targeting resistant hypertension, lowers both systolic and diastolic pressures and, when added to AF ablation, significantly improves arrhythmia‐free survival. Across mixed‐etiology cohorts, 69% of patients receiving combined therapy remained free from AF at 12 months, compared to 29% with ablation alone (Pokushalov et al. 2012; Conen et al. 2009). In paroxysmal AF patients with suboptimal HTN control, arrhythmia‐free rates rose to 72.1% versus 56.5% after ablation alone (Steinberg et al. 2020). Pokushalov et al. further demonstrated that this synergistic approach is most effective in those with severe drug‐resistant hypertension (BP ≥ 160/100 mmHg) and persistent AF, achieving the greatest reduction in recurrence compared with patients having moderate‐resistant HTN (BP ≥ 140/90 and < 160/100 mmHg) (Pokushalov et al. 2014).

## 5 Diabetes

### 5.1 Epidemiology

The Framingham Heart Study first demonstrated that type 2 diabetes mellitus (T2DM) is associated with an elevated risk of incident AF (Benjamin et al. 1994), a finding reinforced by a meta‐analysis of cohort and case–control studies showing a 34% higher AF risk in individuals with diabetes (Huxley, Filion, et al. 2011; Frost et al. 2005). Beyond incident AF, T2DM also predisposes to more persistent arrhythmia: diabetic patients were 1.31 times as likely to present with non‐paroxysmal AF and had 1.32‐fold greater odds of progressing from paroxysmal to non‐paroxysmal AF (Huxley, Filion, et al. 2011; Alijla et al. 2021). In the ADVANCE trial, those with T2DM and AF experienced significantly higher cardiovascular event rates and mortality than T2DM patients without AF (Pallisgaard et al. 2016). A Danish cohort by Pallisgaard et al. further revealed that younger diabetic individuals carry a disproportionately greater AF risk (Pallisgaard et al. 2016). Sex‐specific analyses underscore that poor glycaemic control in T2DM increases AF risk by 35%, with men showing higher absolute risk yet women, particularly those under 55, exhibiting a two‐fold relative increase; diabetic women with stage 5 chronic kidney disease faced a three‐fold elevated risk (Nichols et al. 2009; Krahn et al. 1995). Finally, pharmacologically treated diabetes patients have a 40% higher AF risk versus non‐diabetics, and each additional year of diabetes treatment corresponds to a 3% incremental rise in AF risk, suggesting a dose–response relationship with cumulative hyperglycaemia exposure (Dublin et al. 2010).

### 5.2 Protective Effects of Metformin, TZDs and DPP‐4 Inhibition

Metformin, a first‐line therapy in T2DM, is associated with a reduced risk of new‐onset AF (Chang et al. 2014). Thiazolidinediones (TZDs) confer similar protection: a meta‐analysis showed a 30% lower AF risk in TZD‐treated T2DM patients versus controls (Zhang et al. 2017), and individual cohorts corroborate this finding (Liou et al. 2018). Moreover, in metformin‐treated patients, addition of a DPP‐4 inhibitor further lowers AF incidence compared with add‐on sulfonylureas or other hypoglycaemics (Chang et al. 2017).

### 5.3 Neutral Agents and Insulin‐Related Risk

In contrast, sulfonylureas, acarbose, and glinides do not measurably affect new‐onset AF risk (Liou et al. 2018; Chen et al. 2017). A case–control study likewise found no association between biguanides or TZDs and AF risk, but did identify insulin use as significantly increasing AF incidence (Chen et al. 2017). Although the ORIGIN trial did not find a direct link between insulin therapy and AF, this may be confounded by greater comorbidity in insulin‐requiring patients (Gerstein et al. 2012).

### 5.4 Impact of Diabetes on Cardioversion and Antiarrhythmic Efficacy

Diabetes impairs both cardioversion success and the efficacy of antiarrhythmic drugs in AF. A meta‐analysis found that higher baseline glycated hemoglobin levels in diabetic patients predict increased AF recurrence after catheter ablation, underscoring the importance of glycaemic control for optimizing post‐ablation outcomes (Anselmino et al. 2015). Moreover, diabetic patients not receiving β‐blockers are more prone to cardioversion failure following ablation, whereas those on β‐blocker therapy exhibit improved conversion rates (Soran et al. 2018; Ebert et al. 2018). In animal models, diabetes likewise diminishes the antiarrhythmic effectiveness of flecainide (Ito et al. 2006).

### 5.5 Diabetes and AF Symptom Perception and Severity

Patients with both diabetes and AF experience more severe disease, prolonged AF episodes, higher mortality, and increased hospitalisations (Echouffo‐Tcheugui et al. 2017). Paradoxically, diabetic autonomic neuropathy may blunt cardiac nerve sensitivity, leading to reduced symptom awareness (Rizzo et al. 2015). Some investigations report that individuals with diabetes perceive AF symptoms less intensely than non‐diabetic counterparts (Sugishita et al. 2003). In a cohort of 2411 AF patients, diabetics, despite worse quality of life and a greater comorbidity burden, reported milder symptoms, with no clear link to AF progression (Bano et al. 2021). Similarly, the FRACTAL registry of new‐onset AF found no significant association between diabetes and symptom burden (Reynolds et al. 2006).

## 6 Obstructive Sleep Apnoea

### 6.1 Epidemiology

Sleep apnoea, classified primarily as obstructive sleep apnoea (OSA) or central sleep apnoea (CSA), exhibits a lower prevalence in CSA compared to OSA. In the Outcomes of Sleep Disorders in Older Men study, CSA showed a strong association with AF, whereas OSA and resultant hypoxaemia were linked to broader cardiovascular events (Mehra et al. 2009). Among adults aged 30–65 years, OSA affects roughly 16% of men and 5% of women, and remains undiagnosed in about 5% of the population (Bounhoure et al. 2005; Young et al. 2002).

The prevalence of OSA among AF patients greatly exceeds that in general cardiology cohorts. Up to 49% of individuals with AF have OSA, compared with 32% in non‐AF cardiology patients, highlighting a particularly strong link between sleep‐disordered breathing and AF (Gami et al. 2004). In the Sleep Heart Health Study, AF occurred in 4.8% of those with sleep‐disordered breathing versus 0.9% without, a more than fourfold difference (Mehra et al. 2006). Even among relatively young, otherwise healthy patients with paroxysmal or persistent AF, 62% had OSA compared to 38% of non‐AF controls, and those with more frequent or sustained AF episodes exhibited higher rates of sleep‐disordered breathing than patients with infrequent paroxysms (Stevenson et al. 2008).

Nocturnal AF is markedly more common in OSA, occurring in 3%–5% of OSA patients versus about 0.4%–1% of the general populace (Mehra et al. 2009; Monahan et al. 2009). Moreover, the odds of an arrhythmic event spike eighteen‐fold within 90 s of an apnoeic episode in sleep‐disordered breathing, and AF episode frequency tends to increase immediately following respiratory events (Monahan et al. 2009).

### 6.2 CPAP and AF Recurrence in OSA

Continuous positive airway pressure (CPAP) markedly reduces AF recurrence in patients with obstructive sleep apnoea (OSA). Untreated OSA patients experience an 82% AF recurrence rate within 1 year of cardioversion—nearly double the rate seen in those treated with CPAP (Monahan et al. 2012; Kanagala et al. 2003; Bavishi et al. 2019). In CPAP‐treated OSA patients, AF‐free survival is 71.9% versus 36.7% in non‐CPAP patients (Fein et al. 2013). Moreover, CPAP lowers recurrence regardless of ablation: one study reported up to a 31% increased risk of recurrence in untreated OSA patients post‐ablation (Shukla et al. 2015), and another found a 44% lower recurrence risk with CPAP versus no CPAP (Qureshi, Nasir, et al. 2015). Lower nocturnal oxygen saturation in untreated OSA further predicts recurrence, underscoring the need for adequate nightly CPAP use (Kanagala et al. 2003).

### 6.3 Mechanistic Insights: Atrial Remodeling

The SLEEP‐AF trial demonstrated that CPAP reverses AF‐associated atrial remodeling in OSA patients, thereby improving rhythm outcomes (Nalliah et al. 2022). This structural benefit provides a pathophysiological rationale for integrating CPAP into AF management when OSA coexists.

### 6.4 Limitations and Negative Trials

Not all studies confirm CPAP's benefit for AF. The SAVE trial found no significant difference in new‐onset AF between CPAP and control groups in moderate‐to‐severe OSA (Caples et al. 2019), and a small prospective study similarly reported no impact on recurrence (Caples et al. 2019). Suboptimal adherence, with only about half of AF patients persisting with CPAP, likely biases many analyses that rely on self‐reported use (Weaver and Grunstein 2008; Patel et al. 2010). In paroxysmal AF, CPAP failed to reduce AF burden or improve daytime sleepiness and quality of life, although participants had normal left ventricular function, which may limit clinical relevance (Traaen et al. 2021).

### 6.5 Alternative OSA Therapies and AF

Other OSA treatments remain under investigation for AF prevention. Phrenic nerve stimulation halves respiratory events and normalizes breathing patterns in central sleep apnoea, but its effect on AF has yet to be established (Ponikowski et al. 2012). Positional therapy reduces the apnoea–hypopnoea index less effectively than CPAP, though it achieves higher patient adherence, making it a potential adjunct in selected cases (Srijithesh et al. 2019).

### 6.6 Lifestyle Risk‐Factor Management

Moderate alcohol intake, particularly before sleep, significantly increases both the frequency and duration of OSA episodes, making alcohol screening essential in AF patients with suspected sleep‐disordered breathing (Scanlan et al. 2000). Obesity is strongly linked to OSA; weight loss, whether via lifestyle modification or bariatric surgery, can reduce the frequency and severity of apnoeic events and thereby mitigate AF risk (Araghi et al. 2013).

### 6.7 Screening for OSA in Nocturnal AF

Patients with predominantly nocturnal AF should be evaluated for OSA before pursuing rhythm control. While polysomnography remains the gold standard for diagnosing sleep‐disordered breathing, its cost and limited availability favor alternative modalities: home polygraphy as a screening test, and overnight pulse‐oximetry to estimate the Oxygen Desaturation Index (ODI). When combined with the Apnoea–Hypopnea Index (AHI), ODI measurement offers high sensitivity and specificity for moderate to severe OSA and is more practical for repeated assessments (Linz, Kadhim, et al. 2018).

### 6.8 Symptom Variability, Diagnostic Limitations, and Longitudinal Monitoring

Excessive daytime sleepiness does not reliably reflect the presence or severity of OSA, and many AF patients remain asymptomatic despite significant sleep‐disordered breathing (Albuquerque et al. 2012; Kadhim et al. 2019). Moreover, AHI alone fails to capture nocturnal hypoxaemia burden, night‐to‐night variability, or event clustering (Linz, Baumert, et al. 2018). In AF management, especially when considering cardioversion or ablation, evaluating nightly SDB patterns over time may better predict AF burden than a single‐night study (Desteghe et al. 2018; Linz, Baumert, et al. 2019).

## 7 Alcohol

### 7.1 Epidemiology of Alcohol Intake and AF Risk

Excessive alcohol intake is strongly linked to increased AF risk, whereas low consumption levels may exert a modest protective effect against incident AF (Bell et al. 2017). Animal studies using porcine models confirm that high blood alcohol concentrations increase the likelihood of new‐onset AF, but even low‐to‐moderate intake shows a positive correlation with AF development (Anadon et al. 1996). Consumption exceeding 14 standard drinks per week triples the risk of progression from paroxysmal to persistent AF (Ruigómez et al. 2005).

### 7.2 Dose–Response and Beverage Type

Meta‐analyses show an 8% rise in AF risk for every six drinks per week, supporting a clear dose–response relationship (Kodama et al. 2011). Csengeri et al. found incident AF linked to intake as low as 1.2 drinks daily (Csengeri et al. 2021). Larsson et al. demonstrated that moderate (1–3 drinks/day) and high (> 3 drinks/day) alcohol intake both elevate AF incidence, with an 8% increase per daily drink, and found a positive link for wine and liquor but not beer (Larsson et al. 2014). However, other studies report conflicting findings, with Frost et al. noting no difference in AF risk by beverage type (Frost and Vestergaard 2004).

### 7.3 Thresholds, Sex Differences and Recurrence

Across studies using grams per day, consumption above 20 g/day in men consistently raises AF risk, a pattern not seen in women (Frost and Vestergaard 2004). The Framingham study found that up to 36 g/day raises AF risk by 34%, with intake below this showing no clear effect (Djoussé et al. 2004). Similarly, Koskinen et al. identified higher AF risk above 30 g/day but not at lower levels (Koskinen et al. 1987). Gender‐specific analyses show that women consuming up to two drinks daily have no increased AF risk, while intake beyond two drinks shows a slight association (Conen et al. 2008). In men, risk rises with consumption of at least 35 drinks per week; in contrast, women showed no increased risk even at 21 or more drinks per week (Mukamal et al. 2005). Among patients with pre‐existing AF, alcohol consumption below 23 standard drinks per week in men and 12 in women was still associated with increased AF recurrence compared with non‐drinkers (Planas et al. 2006).

### 7.4 Heavy and Binge Drinking and Incident AF Risk

Heavy alcohol consumption (≥ 40 standard drinks/week) confers a greater AF risk than obesity or hypertension (Sano et al. 2014). Binge drinking—defined as > 5 drinks on one occasion—also elevates AF risk to levels comparable with moderate habitual intake (≤ 21 drinks/week), particularly in elderly patients with pre‐existing cardiovascular disease (Liang et al. 2012). Unlike the J‐shaped curve seen in other outcomes, AF risk increases linearly with total alcohol intake (Larsson et al. 2014; Kodama et al. 2011; Samokhvalov et al. 2010). Meta‐analyses confirm that even former binge drinkers retain a heightened AF risk despite cessation (Larsson et al. 2014; Kodama et al. 2011; Samokhvalov et al. 2010).

### 7.5 Alcohol and Post‐Ablation Arrhythmia‐Free Survival

In patients undergoing AF ablation, consumption above sex‐specific thresholds (> 7 drinks/week in women; > 14 in men) reduces 1‐year arrhythmia‐free survival, with non‐drinkers faring best (Qiao et al. 2015). A ≥ 1% reduction in alcohol intake correlates with a 37% drop in recurrence rates, and among those drinking ≥ 120 g/week at baseline, reducing intake yields a 44.5% lower recurrence rate (Takahashi et al. 2021). Paroxysmal and persistent AF patients benefit most from alcohol reduction, whereas longstanding persistent AF patients show similar outcomes to controls (Takahashi et al. 2021).

### 7.6 Impact of Alcohol Abstinence

Complete abstinence from alcohol among regular drinkers cuts 6‐month recurrence rates to 53% versus 73% in controls and reduces moderate–severe symptoms from 32% to 10%. Abstainers also experience weight loss, which may further decrease AF recurrence (Voskoboinik et al. 2020).

## 8 Cardiorespiratory Fitness

### 8.1 Cardiorespiratory Fitness and Incident AF

Higher cardiorespiratory fitness (CRF) and greater physical activity are linked to lower AF incidence and improved outcomes in those who develop AF. In the Kuopio Ischaemic Heart Disease study, maximal oxygen uptake exhibited a non‐linear inverse relationship with new‐onset AF across a defined fitness range (Khan et al. 2015). Similarly, the HUNT3 cohort demonstrated that AF patients with elevated CRF and activity levels faced reduced long‐term cardiovascular and all‐cause mortality (Garnvik et al. 2020).

### 8.2 Exercise Intensity and AF Risk

A U‐shaped association exists between exercise intensity and AF. In the Cardiovascular Health Study of individuals aged ≥ 65 years, moderate exercise reduced incident AF by 28% compared to inactivity, whereas high‐intensity exercise offered no additional benefit (Mozaffarian et al. 2008). Endurance athletes with very high CRF engaging in strenuous training actually showed an increased AF risk versus non‐athletes (Abdulla and Nielsen 2009; Elosua et al. 2006).

### 8.3 Vigorous Exercise, Age, and AF

Among younger and middle‐aged men, frequent vigorous exercise raises AF risk. The Physician's Health Study found a 20% higher incident AF rate in men performing vigorous exercise five to seven times weekly over 3 years, with this association diminishing with age and disappearing in men over 50 years (Aizer et al. 2009).

### 8.4 Cardiorespiratory Fitness in Obesity and Post‐Ablation Patients

In obese AF patients, CRF, expressed as metabolic equivalents (METs), which convert treadmill test duration (Bruce Protocol) into a value relative to age‐ and sex‐predicted peak capacity, strongly predicts arrhythmia outcomes. In the CARDIO‐FIT study, baseline METs were measured and then re‐assessed at follow‐up: each 1‐MET increase corresponded to a 20% lower AF recurrence risk, while those achieving ≥ 2‐MET gains alongside ≥ 10% weight loss saw a two‐fold reduction. Notably, 61% of initially symptomatic patients no longer required ablation or antiarrhythmic therapy (Pathak, Elliott, et al. 2015). Broader analyses mirror these findings, showing a 7% AF risk reduction per 1‐MET gain, with obese individuals deriving greater proportional benefit than non‐obese peers (Qureshi, Alirhayim, et al. 2015). In post‐ablation cohorts followed for 32 months, higher CRF similarly correlated with fewer AF recurrences, reduced arrhythmia‐related hospitalisations and repeat procedures, lower antiarrhythmic use, and decreased mortality (Donnellan, Wazni, Harb, et al. 2020).

## 9 Pathophysiology

### 9.1 Obesity

#### 9.1.1 Epicardial Adipose Tissue

Epicardial adipose tissue (EAT), located between the visceral pericardium and myocardium microcirculation, contributes to AF via paracrine inflammation, fibrosis, and autonomic modulation. Increased EAT volume correlates with persistent AF and elevated cytokines such as MCP‐1, IL‐1, IL‐6, soluble IL‐6 receptor and TNF‐α which promote local inflammation (Gaborit et al. 2015; Monti et al. 2020; Marchington and Pond 1990; Iacobellis et al. 2009; Haemers et al. 2017; Kusayama et al. 2016; Chen and Turker 2012; Goeller et al. 2018; Liu et al. 2020). Pro‐fibrotic mediators in EAT, such as YKL‐40 and CTGF, drive collagen deposition in the left atrium, creating conduction block and delay that facilitate re‐entrant circuits (Everett and Olgin 2007; Wang, Shen, et al. 2018; Wang, Xi, et al. 2018; Abe et al. 2018; Shaihov‐Teper et al. 2021). EAT also houses the ganglionic plexus that alters autonomic tone and, through oxidative stress and disrupted Ca^2+^ handling, furthers electrical remodeling to sustain AF (Kim et al. 2005; Voigt et al. 2014).

#### 9.1.2 Adipose and Systemic Inflammation

Obesity induces macrophage infiltration into adipose tissue, a process reversed by weight loss (Xu et al. 2003; Weisberg et al. 2003), and shifts the immune profile toward pro‐inflammatory CD8^+^ T cells, whereas lean individuals have more regulatory CD4^+^ T cells that suppress inflammation (Feuerer et al. 2009; Nishimura et al. 2009; Gordon and Martinez 2010). AF patients exhibit elevated CRP, HSP‐β1, IL‐6, IL‐8, and TNF‐α, with CRP prospectively predicting incident AF (Chang et al. 2022; Guo et al. 2012; Aviles et al. 2003; Conen et al. 2010; Dernellis and Panaretou 2001). TNF‐α alters connexin‐40 expression, activates myofibroblasts via TGF‐β, and upregulates MMP‐2/MMP‐9, promoting fibrosis (Liew et al. 2013; Sawaya et al. 2007). IL‐6 drives electrical remodeling and early fibrosis through STAT3 signaling, impairs gap‐junction formation, and, when blocked, reverses Ca^2+^ prolongation and reduces AF incidence (Liu et al. 2021; Chen et al. 2020; Liao et al. 2021; Lazzerini et al. 2019).

#### 9.1.3 Oxidative Stress

Obesity‐related oxidative stress in adipose tissue generates reactive oxygen species (ROS) that increase late Na^+^ currents, provoke early depolarisations, and dysregulate L‐type Ca^2+^ channels and RyR2, prolonging action potential duration and triggering arrhythmias (Suzuki et al. 2003; Sun et al. 2020; Samman Tahhan et al. 2017; Pezhouman et al. 2018; Yoo et al. 2018). ROS also stimulate atrial fibroblast proliferation and upregulate pro‐fibrotic factors such as MMP‐9, p38, and c‐Jun, further advancing structural remodeling (Liang et al. 2018).

### 9.2 Hypertension

Hypertension, present in over 60% of AF patients, drives atrial remodeling through mechanical, structural, and electrophysiological pathways (Kallistratos et al. 2018). Chronically elevated blood pressure impairs ventricular systolic–diastolic function, raising left atrial pressure and causing atrial dilatation, a key substrate for AF development (Pluteanu et al. 2015; Fialová et al. 2008). At the cellular level, hypertensive hearts exhibit disrupted Ca^2+^ handling, cardiomyocyte ultrastructural abnormalities, inflammatory cell infiltration, and fibroblast activation, all of which increase arrhythmia susceptibility (Pluteanu et al. 2015; Fialová et al. 2008). Early in hypertension, the left atrium also undergoes electrical alterations—heightened conduction heterogeneity, shortened atrial wavelength, and prolonged AF episodes—further linking elevated blood pressure to AF onset and persistence (Choisy et al. 2007; Lau et al. 2013).

### 9.3 Obstructive Sleep Apnoea

OSA promotes AF through intertwined structural, electrical, and autonomic pathways. Intermittent hypoxia and negative intrathoracic pressures induce sympathetic surges, causing tachycardia, hypertension, and relative atrial ischemia that precipitate diastolic dysfunction and left atrial enlargement (Fung et al. 2002; Alchanatis et al. 2000; Skalidis et al. 2008; Lin et al. 2012). Hypoxic episodes also slow atrial conduction and increase conduction heterogeneity, creating a substrate for re‐entrant arrhythmias (Linz et al. 2012, 2011). Concurrently, prolonged apnoeic pauses enhance neuronal firing in the epicardial ganglionic plexus near the pulmonary veins, facilitating both AF initiation and maintenance (Ghias et al. 2009). Over time, OSA‐driven sympathovagal imbalance and profibrotic signaling culminate in atrial fibrosis, further stabilizing the arrhythmogenic substrate (Skalidis et al. 2008; Lin et al. 2012; Li et al. 2014).

### 9.4 Diabetes

Type 2 diabetes mellitus (T2DM) promotes AF through intertwined structural, electrical, autonomic, and metabolic mechanisms (Russo and Frangogiannis 2016).

#### 9.4.1 Structural Remodeling

Chronic hyperglycaemia elevates reactive oxygen species and suppresses antioxidant defenses, triggering inflammation (↑CRP, TNF‐α) and release of profibrotic factors (TGF‐β, AGEs) that drive atrial fibrosis and dilation (Russo and Frangogiannis 2016; Ziolo and Mohler 2015; Guo et al. 2012; Faria and Persaud 2017; Liu et al. 2012; Kato et al. 2008). Ventricular stiffening and diastolic dysfunction further enlarge the atrium, creating a substrate for re‐entry (Tiwari et al. 2015).

#### 9.4.2 Electrical Remodeling

Diabetes alters atrial conduction, prolonging activation times, increasing electromechanical delay and disrupting connexin‐mediated gap junctions, thereby facilitating arrhythmia initiation (Liu et al. 2012; Chao et al. 2010; Watanabe et al. 2012; De Vos et al. 2009; Demir et al. 2016; Bohne et al. 2019).

#### 9.4.3 Autonomic Remodeling

Cardiac autonomic neuropathy in T2DM skews autonomic balance toward sympathetic dominance, heightening AF susceptibility and contributing to silent episodes (Kuehl and Stevens 2012; Rizzo et al. 2015).

#### 9.4.4 Glycaemic and Adipokine Influences

Fluctuating glucose levels exacerbate risk: hypoglycaemia triggers sympathetic surges, while hyperglycaemia augments oxidative stress and fibrosis (Liu et al. 2012; Ko et al. 2018; Monnier et al. 2006). Epicardial fat–derived adipokines compound these effects by promoting local inflammation, oxidative injury and connective tissue deposition (Bohne et al. 2019).

### 9.5 Alcohol

Alcohol contributes to AF through acute ion‐channel modulation, chronic structural remodeling, and autonomic dysregulation (Yan et al. 2018; Zhang et al. 2020; Zhao et al. 2015).

#### 9.5.1 Acute and Intermediate Arrhythmogenic Effects

Within hours of ingestion, alcohol shortens atrial action potentials and increases refractoriness dispersion by upregulating acetylcholine‐sensitive potassium channel, enhanced repolarisation, and reducing L‐type Ca^2+^ and Na^+^ current density (Zhang et al. 2020; Anadon et al. 1996; Zhao et al. 2015; Chen et al. 2004). Binge episodes further enhance T‐type Ca^2+^ channel expression, potentially precipitating an arrhythmia known as “holiday heart syndrome” (Wang et al. 2020). Clinically, acute intake reduces atrial refractory periods, slows intra‐atrial conduction, prolongs His–ventricular intervals, and lengthens sinus‐node recovery, while P‐wave duration and electromechanical delay both increase after heavy drinking (Gould et al. 1978; Greenspon and Schaal 1983; Steinbigler et al. 2003).

#### 9.5.2 Chronic Alcohol Consumption and Atrial Cardiomyopathy

Sustained consumption drives structural and functional atrial changes. In animal models, prolonged exposure decreases myofilament Ca^2+^ sensitivity, impairs inotropic response, slows conduction, shortens refractoriness, and increases its dispersion (Zhang et al. 2020; Piano et al. 1999). Human studies reveal that each standard drink raises the odds of low‐voltage atrial regions by 10% during pulmonary vein isolation, and drinkers exhibit more complex potentials, slower conduction velocities, and enlarged left atrial diameters, all markers of atrial mechanical dysfunction that predict AF (McManus et al. 2016; Voskoboinik et al. 2018; Voskoboinik, Costello, et al. 2019; Singh et al. 2013; Voskoboinik, Wong, et al. 2019; Qiao et al. 2015).

#### 9.5.3 Autonomic Modulation

Alcohol acutely activates both sympathetic, reducing heart rate variability (HRV), and vagal pathways (shortening refractoriness), with simultaneous discharges serving as potent AF triggers (Linz, Elliott, et al. 2019; Voskoboinik et al. 2021; Weise et al. 1986; Brunner et al. 2021). During a “hangover,” atrial ectopy rises and HRV remains suppressed, fostering paroxysmal episodes during vagal periods such as rest or sleep (Brunner et al. 2017). In hypertensive individuals, alcohol amplifies pressor responses, further elevating blood pressure and AF risk (Hering et al. 2011).

### 9.6 Cardiorespiratory Fitness

Mechanical stretch, inflammation‐driven fibrosis, and autonomic–ectopic triggers converge to transform adaptive “athlete's heart” into a pro‐fibrillatory state (Sanz‐de la Garza et al. 2016).

#### 9.6.1 Mechanical Overload and Atrial Remodeling

Chronic high‐intensity exercise imposes sustained volume and pressure overload on the atria, particularly the left atrium and pulmonary veins, leading to bi‐atrial enlargement and wall stress (Turagam et al. 2015; Sanchis‐Gomar et al. 2017). In elite athletes, left atrial enlargement affects ~20% and correlates with cumulative training hours (24% for < 1500 h; 40% for 1500–4500 h; 83% for > 4500 h) (Pelliccia et al. 2005; Wilhelm et al. 2011). Persistent stretch induces myocyte micro‐trauma, apoptosis, and upregulation of profibrotic pathways (TGF‐β, MAPK), initiating structural remodeling (Turagam et al. 2015; Sanchis‐Gomar et al. 2017).

#### 9.6.2 Inflammation and Fibrosis

Mechanical injury and exercise‐induced oxidative stress generate local inflammation and activate collagen turnover via PI3K‐Akt and renin–angiotensin signaling (Swanson 2006; Carbone et al. 2017; Guasch and Mont 2017). Biomarkers of myocardial fibrosis (CITP, PICP, TIMP‐1) are elevated in veteran endurance athletes versus controls (Lindsay and Dunn 2007). Cardiac MRI studies confirm late gadolinium enhancement in 50% of veteran athletes, with increased years of training and marathons predicting the prevalence of fibrosis (Wilson et al. 2011).

#### 9.6.3 Autonomic Modulation and Ectopic Triggers

Endurance training shifts autonomic balance toward high vagal tone interspersed with adrenergic surges, shortening atrial refractory periods and increasing dispersion to favor re‐entry (Sanz‐de la Garza et al. 2016; Mont et al. 2009; Stergiou and Duncan 2018). Repetitive exercise also raises pulmonary‐vein and ventricular ectopy, triggers for paroxysmal AF, although data on atrial ectopy remain mixed. These electrical perturbations on a fibrotic, dilated atrium readily initiate and maintain AF (Mont et al. 2009).

#### 9.6.4 Low CRF

Conversely, low CRF and sedentary behavior foster obesity, hypertension, and diastolic dysfunction, which similarly elevate atrial pressure, promote low‐grade inflammation, and cause interstitial fibrosis, creating the same arrhythmogenic substrate seen in highly trained athletes (Sanchis‐Gomar and Lavie 2022).

## 10 Conclusion

Atrial fibrillation is increasingly driven by modifiable cardiometabolic and lifestyle exposures that drive atrial stretch, inflammation, oxidative stress, fibrosis and autonomic imbalance, creating a substrate that favors ectopy and re‐entry. Across the literature, addressing these upstream drivers is consistently associated with lower arrhythmia burden and more durable rhythm‐control outcomes. Although the relative contribution and optimal timing of individual interventions remain uncertain, integrating structured risk‐factor management into AF care targets the underlying substrate and improves long‐term outcomes.

## Author Contributions

Chamath Jagoda conceived and designed the study, acquired and analysed the data, and drafted and approved the final manuscript.

## Funding

The author has nothing to report.

## Disclosure

Authorship declaration: All authors meet the authorship criteria and are in agreement with the manuscript.

## Conflicts of Interest

The author declares no conflicts of interest.

## Data Availability

Data sharing is not applicable to this article as this is a narrative review and no new datasets were generated or analyzed.
